# Estimation the medical cost of multiple sclerosis in Iran; 2019–2020

**DOI:** 10.1186/s12913-022-07551-z

**Published:** 2022-02-02

**Authors:** Mina Asadollahi, Ali Darvishi, Amirreza Azimi, Majid Annabi, Zahra Jafariazar, Ramin Heshmat

**Affiliations:** 1 Pharmacoeconomic and Pharmaceutical Management Department, Faculty of Pharmacy and Pharmaceutical Sciences, Tehran Medical Sciences-Islamic Azad University, Tehran, Iran; 2 Chronic Diseases Research Center, Endocrinology and Metabolism Population Sciences Institute, Tehran University of Medical Sciences, No 10, Jalale-Al-Ahmad Ave, Chamran Highway, Tehran, 1411713137 Iran; 3 Department of Health Management and Economics, School of Public Health, Tehran University of Medical Sciences, Tehran, Iran; 4 Multiple Sclerosis Research Center, Neuroscience Institute, Tehran University of Medical Sciences, Tehran, Iran; 5 Pharmaceutics Department, Faculty of Pharmacy and Pharmaceutical Sciences, Tehran Medical Sciences-Islamic Azad University, Tehran, Iran

**Keywords:** Multiple sclerosis, Economic burden, Medical cost, Iran

## Abstract

**Background:**

Due to the high and increasing economic burden of chronic diseases, including Multiple sclerosis (MS), we aimed to investigate the medical cost of MS in Iran.

**Methods:**

This is a descriptive cross-sectional study which conducted using comprehensive national prescription data from Iran’s Health Insurance Organization (IHIO) and rehabilitation data from Ministry of Health and Iran Welfare Organization. The time period considered for this study was 2019–2020. In order to calculate the medical cost of MS, the cost-of-illness (COI) method was used based on the prevalence-based approach and the cost of medications, determining and diagnosing the MS risk, follow-up and rehabilitation was estimated.

**Results:**

The total medical cost of MS in Iran in 2019–2020 was estimated at $238,124,160, which medications and rehabilitation services had the largest share in the medical cost of MS in Iran with 80 and 19%, respectively, and the cost share of determining and diagnosing of the disease risk accounted for about less than 1%. The total medication cost was estimated to be equal to $192,298 thousand. The total cost of determining and diagnosing of the MS risk was estimated at $348,574 and the total cost of rehabilitation services for all MS subgroups in 2019–2020 was estimated at $45,477,205.

**Conclusions:**

Results of calculating the medical cost of MS in Iran in 2019–2020 showed a significant burden on the Iranian health care system and society, among which the medication cost had the largest share, which requires serious attention of health system policymakers.

## Background

Multiple sclerosis (MS) is an inflammatory disease of the central nervous system. MS has an unknown etiology and is therefore classified as a complex disease [1]. It is the most common neurological disease among young adults [2], and according to latest studies, as of 2020, 43.95 per 100,000 population worldwide were affected by this disease [3]. In Iran, the prevalence of MS in 2017 was estimated 62,000 populations and age-standardized rate per was 69.5 per 100,000 population [4].

MS clinically divided into several subgroups, that the most important of which are Relapsing-Remitting (RR) [5], primary progressive (PP) [6] and secondary progressive (SP) [7]. According to available statistics, about 85% of MS cases in the world are relapsing-remitting [8]. The most common type of MS in Iran, which accounts for approximately 88% of cases, also is the RR type. RRMS may become a SPMS type after 10 to 15 years [9].

The disease affects a wide range of men and women, mainly young people, such that the average age of disease onset is estimated to be about 30 years old [10]. There are also various evidences in terms of mortality and disability of MS patients. According to the latest review studies in 2016, 18,932 people worldwide died due to MS, and the number of disability-adjusted life years (DALYs) was estimated [11] at 115,148 units [12]. In Iran DALYs was estimated at 29.1 per 100,000 population in 2017 [4].

There are variety of interventions are used to treat MS patients and includes preventive and supportive cares, rehabilitation and symptoms management [11, 13]. The use of disease modifying therapies (DMTs) is a common type of interventions. These Medications are used to change the course of the disease, treat relapses, and manage symptoms [14]. In addition to these interventions, rehabilitation services also ultimately leads to reducing fatigue and improving the patients’ quality of life.

Due to the debilitating nature of this chronic and progressive disease which affects people during his productive life years, the economic burden of MS is considerable [15], which a large part of this economic burden is borne by the patients themselves [11]. Based on latest studies the catastrophic health expenditure of MS patients in North-west of Iran was estimated at 54 and 44% of household with MS patients experienced poverty because of out of pocket payments (OOP) [16].

It also has high economic cost for society, the health care system and the family. This cost share is especially important for insurance companies. Since nearly 80% of the medications used in Iran are covered by insurance organizations, it increases the financial burden imposed on insurance organizations. Many special patients, including MS (90%), are exempt from the deductible. Therefore, this type of disease puts more financial pressure on insurance organizations and the country’s health system. In addition to the increase in the covered population, increase in the average number of visits, increase in drug prices, gradual elimination of drug subsidies, the entry of new medications into the market and the use of expensive drugs and lack of strict control of prescriptions should be considered. This indicates a double burden on insurance companies to cost reimbursement [17].

The economic burden of diseases is a valuable indicators in health systems, so the economic burden studies is very necessary and important to identifying the economic dimensions of the disease current situation in order to applying the best strategies. Based on the explanations provided regarding the various dimensions of the issue, and considering that a comprehensive study on estimating the costs and economic burden of MS in Iran has not been conducted, the present study aimed to measuring the total MS medical cost divided to different sectors and investigating OOP and insurance share of MS medications in Iran in 2019–2020 based on comprehensive national data. Considering the major share of medical cost in total MS economic burden and calculating different of its dimensions, present study can provide good evidence to understand the overall dimensions of the issue and also identification the best strategy to control the effects of the disease in the future. Also, considering that the country’s health system and insurance organizations are directly affected by this issue, results of present study can be effective in applying more detailed rules and guidelines to promote efficiency and equity in this regard.

## Methods

### Study design

This is a descriptive cross-sectional study that aimed to investigate the medical cost of MS in Iran. The time period considered for this study was 2019–2020.

In order to calculate the cost of MS, the cost-of-illness (COI) method was used based on the prevalence-based approach and the bottom-up method. In the COI approach, costs are divided into three groups: direct medical cost, non-medical direct cost, and cost of lost production [18].

Direct medical cost components included payments for determining and diagnosing of the disease risk and treatments, admission of patient, hospitalization, prescriptions, follow-ups, and rehabilitation services. Costs included patient transportation and accommodation cost, food cost or charges for setting appointments refer to non-medical direct cost [19, 20] and the cost of lost production includes the cost of premature death or the cost of losing production due to incapacity for work [18].

Due to the fact that the main economic burden related to MS due to the nature of the disease is allocated to medical expenses, in present study only the cost of different medical dimensions of disease has been estimated and the calculation of non-medical dimensions has been omitted. Medical costs in present study include the cost of determining and diagnosing the MS risk, medications, follow-up and rehabilitation.

### Participants and data gathering

The data sources used for this study consisted of two parts. For calculating medications and diagnosis tests and screening costs, prescriptions records of the Iran Health Insurance Organization (IHIO) was used and in order to rehabilitation calculations, the comprehensive data from the Iran’s ministry of health deputy of treatment and Iran welfare organization data were used.

#### IHIO and data used for calculating medications and diagnosis tests costs

IHIO is the largest basic health insurance in Iran, which started its activities in 2012 and was created to concentrate all health insurance affairs through the amalgamation of several health insurance funds and currently covers more than 41 million people in the Iran. Also, covers patients with special diseases included about 50% of MS patients in the country in 2019–20. For this reason, in present study, the prescription record database of this organization was used, which can provide a relatively accurate estimate of MS patients in the country.

It should be noted that co-payment for MS medications and services in IHIO is different. In the case of medications, this rate varies between 5 and 30%, and for inpatient services in governmental centers, it varies from 5 to 10%.

Due to the fact that there was no separate database for MS patients, the prescriptions for MS patients were analyzed based on MS-related drug information, radiology information, and tests data, and finally disaggregated. Therefore, at first, prescription drugs for MS patients were extracted based on treatment protocols, guidelines and experts panel, and all prescriptions records containing these drugs were extracted from IHIO databases.

The annually pharmaceutical statistics of Iran’s Food and Drug Administration data was also used to correct deficiencies in some insurance prescription data in order to medications. This data is a collection of medication purchases and sales information by summarizing the information submitted by companies. In present study, we used 2019–2020 pharmaceutical statistics which was available on the FDA website.

Based on primary review of IHIO prescription records, overall 34,964 MS patients received services was covered by IHIO in 2019–20. On the other hand, according to the announcement of the Iranian MS association and the Ministry of Health, there are about 70,000 MS patients in the Iran in 2019–20. Accordingly, the data and analysis of present study include 49.94% (≈50%) of the population of MS patients in Iran. For this reason, to estimate the total cost of MS patients in the medication and diagnostic services sectors, it was estimated to double the calculations derived from IHIO data.

#### Data source for rehabilitation services

The comprehensive data of the patients from the Iran’s ministry of health deputy of treatment and Iran welfare organization were used to calculate the medical cost of MS rehabilitation services. Due to the lack of insurance coverage of MS rehabilitation services by the main basic insurances including IHIO, the data related to rehabilitation services could not be extracted from the insurance prescription records and we used the data of the Ministry of Health of total in need patients.

### Data analysis

After extracting the specific MS patients’ prescriptions, the cost share and the number of patients receiving medications were extracted. Based on this first all medications received by MS patients and the number of patients receiving medications was determined, then the total medication cost, the share of IHIO cost and the amount of OOP of each medication were calculated separately. Also the components of diagnosis tests and screening services including radiology and laboratory tests was extracted and costing was conducted.

To calculate the medical cost of MS rehabilitation, the number of patients in different groups of disease severity who underwent rehabilitation services in 2019–20 was extracted and the costing was conducted based on the unit cost used in each intervention. Different groups of diseases including Primary Progressive (PPMS), Secondary Progressive (SPMS) and Non Progressive and the Expanded Disability Status Scale (EDSS) were categorized and the services received in each group were determined according to the Iran’s Ministry of Health standards for MS rehabilitation services. Costing steps were conducted based on bottom-up approach and in consultation with a specialized team and calculated based on public tariffs of the Iran’s Ministry of Health in 2019–20. Rehabilitation services included hospitalization, physiotherapy, speech therapy, occupational therapy and monitoring and maintenance.

All steps including data collection, monitoring and segration, analysis and extraction of results were performed using Stata 14 and Excel 2016 software.

## Results

Findings from calculations of the medical cost of MS patients in Iran for 2019–20 are presented in the form of 3 separate sections including the cost of MS medications, the cost of determining and diagnosing the risk of disease and the cost of rehabilitation services.

### Cost of MS medications

Findings of the calculations of MS medications can be seen in Table 1. Accordingly, the estimation of general and specific medications used, the number of patients received medications (patients covered by IHIO), the total medication cost, the amount of OOP payment by patients and the cost share of IHIO was conducted. Also, the total medication cost for MS patients in the whole country was estimated by doubling the total cost of patients covered by IHIO.

**Table 1:** Total Medication Cost of Patients with MS in 2019–20

Drug Generic Name	Number of Patients received MS medications (IHIO)	Total Drug Cost (1000 $)	Copayment Share (%)	Out of Pocket (1000 $)	IHI Cost share (1000 $)	Cost Per Patient ($)	OOP per Patients ($)	Estimate of Country Total Cost (1000 $)	Share in the Total Cost (%)
Beta-Interferon	21,019	56,280.1	13	7316.4	48,963.7	2677.5	348.08	112,560.3	58.53
Glatiramer Acetate	1434	4251.1	10	425.1	3826.06	2964.5	296.4	8502.3	4.42
Dimethyl Fumarate	8779	7546.4	30	2263.9	5282.5	859.6	257.8	15,092.9	7.84
Fingolimod	7333	7585.4	5	379.2	7206.1	1034.4	51.7	15,170.8	7.88
Teriflunomide	1553	1540.8	30	462.2	1078.5	992.1	297.6	3081.6	1.60
Rituximab	9506	13,470.7	10	1347.07	12,123.7	1417.08	141.7	26,941.5	14.01
Natalizumab	724	5459.05	10	545.9	4913.1	7540.1	754.01	10,918.1	5.67
Cyclophosphamide	192	6.37	10	0.63	5.73	33.1	3.3	12.7	0.006
Mitoxantrone	102	8.88	10	0.88	8	87.1	8.7	17.7	0.009
Total	**50,642**	**96,149.19**	**13.25**	**12,741.51**	**83,407.68**	**17,605.87**	**2159.54**	**192,298.38**	100

As mentioned in methods section the number of MS patients covered by the IHIO was 34,964 in 2019–20. Based on this, out of 9 MS drugs, a total of 50,642 patients received these drugs during the year, which was the highest and lowest share was beta interferon and Mitoxantrone with 21,019 and 102 recipients, respectively. The total cost of the drugs used was estimated at $96,149 thousand, which interferon and rituximab accounting for the largest share and cyclophosphamide for the lowest. Also, the highest out-of-pocket payments went to interferon.

**Fig. 1:**
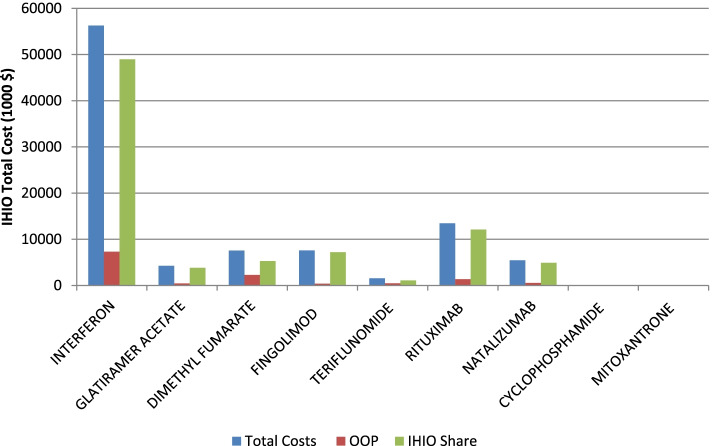
Total Medication Cost of IHIO Patients with Multiple Sclerosis in 2019–20

As mentioned, considering the coverage of about 50% of MS patients by IHIO, the calculated values were doubled to estimate the total medication cost of the total MS population in the country. So the total medication cost of MS patients in Iran was estimated to be equal to $192,298 thousand for the 2019–20. More details of the cost calculations of MS medications could be seen in Table 1 and Fig. 1.

### Cost of determining and diagnosing the MS risk

Findings related to this section are presented in Table 2. The calculations of this section include the cost of specialized periodic visits, the cost of Magnetic resonance imaging (MRI), Cerebrospinal fluid (CSP), Evoked potential (EP) and laboratory tests. Calculations were conducted based on population data covered by IHIO and estimating the total patients in the country.

**Table 2:** Total Cost of Determining and Diagnosing of the MS Risk in 2019–20

Service	Frequency	IHIO Total cost ($)	Country Total Cost ($)	Share in the Total Cost (%)
Specialized Visits	3888	29,808	59,616	17.10
MRI^a^	2991	88,104.29	176,208.59	50.55
CSF^b^	122	3026.26	6052.52	1.73
EP^c^	82	1271.27	2542.55	0.72
*Diagnosis (laboratory* tests)				
CBC	14,662	6799.01	13,598.04	3.90
ALT SGPT	11,702	5697.89	11,395.8	3.26
AST SGOT	11,438	5680.26	11,360.53	3.25
HBS Ag	2334	7607.04	15,214.09	4.36
HCV Ab	2396	6341.90	12,683.82	3.63
ALP	6209	2613.55	5227.101	1.49
TSH	4643	5790.79	11,581.59	3.32
CRP	2857	1003.82	2007.657	0.57
LDL	1500	925.23	1850.462	0.53
Anti-HCV	2090	6368.08	12,736.17	3.65
Hydroxy Vitamin-D	1494	3249.84	6499.69	1.86
Total Cost		174,287.31	348,574.635	

^a^*Magnetic resonance imaging (MRI)*

^b^ *Cerebrospinal fluid (CSP)*

^c^ *Evoked potential (EP)*

The total cost of diagnostic services for the patients covered by IHIO was estimated at $115,000. The highest cost share was for MRI services ($88,104.29), which accounts for about half of the total cost of determining and diagnosing MS risk, given the relatively high frequency of prescriptions. The lowest cost share was for LDL and CRP laboratory tests with $ 925 and $1003, respectively.

**Fig. 2:**
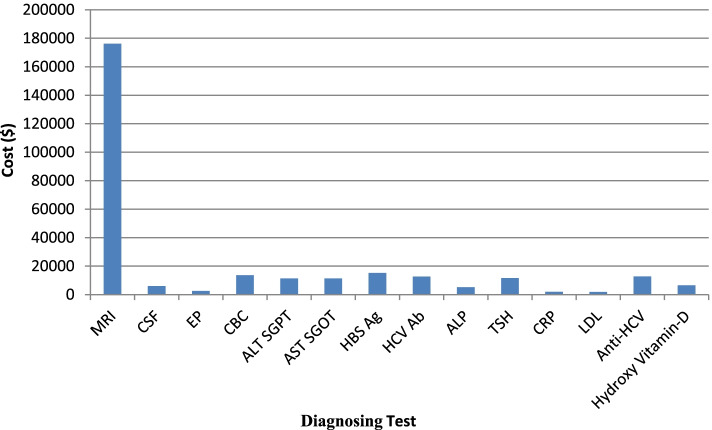
Total Cost of Determining and Diagnosing of the MS Risk in 2019–20

Similar to the previous section, to calculate the cost of the total patients of the whole country, the values ​​obtained from IHIO population were doubled, so the total cost of determining and diagnosing of the MS risk was estimated at $348,574.63 in 2019–2020. The schematic status of each group’s share can be seen in Fig. 2.

### Cost of rehabilitation services

Table 3 shows the findings of the cost of rehabilitation services calculations for all MS patients in Iran in 2019–20. Calculations include inpatient and outpatient rehabilitation costs that have been calculated separately for different groups of patients based on different severity of disease.

**Table 3:** Total Hospitalization and Rehabilitation Cost of Patients with MS in 2019–20

MS Type	Course		Interventions	Patients	Number of Services	Services Unit Cost($)	Cost Per Patients ($)	Total Cost ($)
Primary Progressive (PPMS)	**Three-week course (hospitalization)**		Hospitalization	7024	21	43.47	913	6,413,369
			Physiotherapy	7024	25	5.42	135.52	952,007.9
			Speech Therapy	7024	12	6.71	80.58	566,046.3
			Occupational therapy	7024	20	5.93	118.71	833,942
			***Total Cost***				1247.82	8,765,365
	**One year course (outpatient)**		Physiotherapy	7864	32	5.42	173.47	1,364,289
			Speech Therapy	7024	10	6.71	67.15	471,705.2
			Occupational therapy	6300	22	5.93	130.59	822,723
			***Total Cost***				371.21	2,658,717
Secondary Progressive (SPMS) and Non Progressive	**Three-week course (hospitalization)**		Hospitalization	28,098	7	43.47	304.33	8,551,158
			Physiotherapy	28,098	65	5.42	352.36	9,900,882
			Speech Therapy	4515	18	6.71	120.87	545,830.3
			Occupational therapy	28,098	45	5.93	267.11	750,5478
			***Total Cost***				1044.69	26,503,348
	**One year course (outpatient)**	**EDSS < 3**	Physiotherapy	5017	20	5.42	108.42	544,004.5
			Speech Therapy	4515	3	6.71	20.14	90,971.7
			Occupational therapy	4492	14	5.93	83.1	373,341.7
			***Total Cost***				211.67	1,008,318
		**EDSS (3–5.5)**	Physiotherapy	12,114	26	5.42	140.94	1,707,497
			Speech Therapy	4442	4	6.71	26.86	119,330
			Occupational therapy	10,684	20	5.93	118.71	1,268,405
			***Total Cost***				286.52	3,095,232
		**EDSS (5.5–8.5)**	Physiotherapy	11,182	30	5.42	162.63	1,818,558
			Speech Therapy	4300	5	6.71	33.57	144,399.8
			Occupational therapy	10,864	23	5.93	136.52	1,483,268
			***Total Cost***				332.73	3,446,226
Total Rehabilitation Cost								**45,477,205.5**

According to available data, out of the total number of patients, 34,780 MS patients have undergone rehabilitation services at least once in 2019–20. Accordingly, in the PPMS group of patients, the cost of 3 week inpatient services was estimated at $8,765,365 and the cost of periodic outpatient services was estimated at $2,658,717.

The rehabilitation cost of 3 weeks inpatient services for SPMS and non-progressive patients group was estimated at $26,503,348. The cost of periodic outpatient services in this group varies based on EDSS score levels and has increased according to the severity of the disease. Thus, the cost of outpatient services for these groups was estimated at $1,008,318, $3,095,232 and $3,446,226, for patients with EDSS< 3, 3 < EDSS< 5.5 and 5.5 < EDSS< 8.5, respectively.

Overall, the total cost of rehabilitation services for all MS subgroups in 2019–2020 was estimated at $45,477,205.5.

**Fig. 3:**
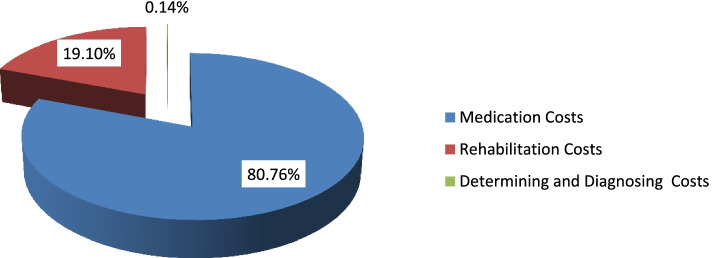
Share of Different Dimensions of Medical Cost of MS Patients in 2019–20

Figure 3 shows the share of different dimensions of medical medical cost of MS patients in 2019–20. Accordingly, it is observed that medications and rehabilitation services have the largest share in the medical cost of MS in Iran with 80 and 19%, respectively. The cost share of determining and diagnosing of the disease risk accounted for about less than 1%. In total, considering the cost of different sectors, the total medical cost of MS in Iran in 2019–20 was estimated at $238,124,160.1.

## Discussion

We aimed to estimate the medical cost of MS in Iran using comprehensive national data of 2019–2020. Findings of the study showed that in general, in the study year, the medical cost of MS, which accounted for the largest share of the total economic burden of the disease [21] was estimated at $238,124,160.1 (10,001 Billion IR Rial), which is a significant figure.

According to the findings, the largest share of the economic burden of MS was allocated to the medications, followed by rehabilitation cost and the cost of determining and diagnosing the MS risk. Blahova et al. (2012) estimated the total annual cost of MS in the Czech Republic in 2012 at around €12,272, which direct medical cost accounting for more than 50% of the total economic burden [22]. In return, Blozik et al. (2017) reported an estimated cost of MS patients in Switzerland of $4 million in 2010, of which 38% was direct medical cost and 67% was non-medical direct cost [23].

The cost of medications used to treat MS was estimated at about 80% of the total medical cost of the disease. Among the 9 specific and general MS medications studied in this study, the highest share of cost with amount equivalent to $112,560 thousand was allocated to beta-interferon 1A and 1B, which were very different from other drugs in this regard. Cyclophosphamide, on the other hand, had the lowest share of the cost at just $12.7 thousand.

Hasnat et al. (2020) in their study, which aimed to examine the increasing cost of MS in several selected countries, reported that the share of pharmaceutical cost in the economic burden of MS was 55% [21]. The findings of a cross-sectional study in Kuwait in 2018 showed that 89% of the medical cost was allocated to medications [24].

Findings of the study on the cost of rehabilitation services showed 19% share of the total medical cost of MS patients, which is a significant amount. According to study by Svendsen et al. (2018) in Norway and Ponzio et al. (2014) in Italy, the cost of rehabilitation services for MS accounted for a significant share of total medical cost [25, 26].

Rehabilitation costs include various services such as physiotherapy, occupational therapy, speech therapy and sub-acute hospitalization, which are used intermittently in different groups of the disease severity to improve the symptoms of the disease and improve patients’ quality of life. Based on the findings of present study, it is clear that the highest cost of rehabilitation services are allocated to groups with more severe disease and patients with higher EDSS scores also create more cost due to the need to repeat more rehabilitation courses. Findings of the Ponzio study in 2014 and Imani et al. (2020) also showed that with increasing disease severity and patients’ EDSS score, the economic burden of the disease and especially rehabilitation cost increases [25, 27].

Among the MS rehabilitation services, physiotherapy services have the largest share of cost in Iran at all levels of the disease. This may be due to higher access to these services as well as the existence of insurance coverage for these services. However, speech therapy and occupational therapy, firstly, are not in optimal position in terms of accessibility and, secondly, they do not have insurance coverage. In this regard, the lack of distributive equity in access to rehabilitation services in Iran is also important.

The point about rehabilitation services is increasing the share of these services in managing the statues of MS patients during the past years, so that with the expansion of rehabilitation service centers, increased physicians’ knowledge of these services and also prove the efficacy of these interventions on improving the patients disease status, especially those with more acute conditions, had increased the use of these services. This is also confirmed by the Hasnat et al. study [21].

Regarding Iran, it should be noted that according to experts and the distribution of patients in terms of disease severity levels, about 70% of MS patients need parts of rehabilitation services in their disease periods, while according to the results of the present study, the utilization rate of rehabilitation services is lower. Accordingly, it should be noted that in case of higher access and proper utilization of these services, the cost of this sector will be much higher. On the other hand, the calculations in this study are based on public sector tariffs, while part of the services are performed in the private sector with higher cost.

Among the MS medical cost, the lowest share was allocated to the determining and diagnosing of disease risk services, including imaging cost, periodic visits and laboratory services. It accounted for about less than 1% of total medical cost. In the Hasnat study, these cost were estimated at 3% of the total direct medical cost of MS [21]. An important point in this category of cost is the share of more than 50% of MRI services among the total cost of determining and diagnosing disease risk services, which should be highly considered. This is due to the higher unit cost of MRI as well as the frequency of repetition of this service during the disease courses in patients with MS.

In general, the results of the study showed that the medical cost of MS in the country is significant and is increasing due to the spread of the disease, increased diagnosed patients and new interventions and medications. According to the the results of a study by Fattahi et al. (2021), the DALYs of MS in Iran has increased from 9098 to 26,183 unit between 1990 and 2017 [4]. On the other hand, the highest amount of these costs, as mentioned, is allocated to direct medical cost, especially medication and rehabilitation services cost, which have a significant burden on the country’s health care system and also uncovered population [15, 27]. The latest studies in Iran confirmed our results. Results of study by Imani et al. (2020) showed that the mean annual cost for MS patients in Iran with the mean EDSS score of 3.14 is $2321.94 [27]. The annual cost incurred divided with mild, moderate and severe levels of disease were 1998.05, 3280.30 and $2856.25, respectively [27].

An increasing trend in the economic burden of this disease is also predicted in the whole world. Nana et al. (2017) in their study, which simulated the epidemiological situation and economic burden of MS in Canada, estimated that by 2031 the total economic burden of MS health services will reach $2 billion [28].

Since the middle of the last decade, with the development of new MS drugs and proving the effectiveness of rehabilitation services for these patients, there have been significant advances in the effectiveness of interventions to improve patients’ quality of life. This shows that the implementation of control policies and careful planning should be done for this group of patients, which in addition to increasing the patients’ quality of life, which in itself reduces the cost of lost production, the burden on the health care system and insurance organizations should also be monitored in order to maintain and complete insurance coverage.

### Limitations

The most important limitation of this study was the lack of access to specific separate prescription and cost data for MS patients in Iran. This problem was largely eliminated by adapting prescriptions to graphs, diagnostic tests, and MS specific drugs for all IHIO prescriptions with the help of clinical specialists. Another point was the lack of access to data from other insurance organizations, including social security insurance, which, considering the sample size of approximately 50% of IHIO data, tried to make a relatively accurate estimate of whole Iran. Another Important limitation was the loose of related data from who were not register in national Plan and IHIO which leads to under estimate of cost calculations.

Regarding rehabilitation services, as mentioned, due to the lack of centralization of registration and the lack of insurance coverage of major services in Iran, the data are probably limited in the number of cases referred to private centers, which does not seem to have a significant share. In this study, lost production cost and non-medical direct cost are not estimated. In future studies, with access to appropriate data, this part of the cost will be calculated, which in addition to the medical cost calculated in this study can provide a more comprehensive picture of the economic burden of MS in Iran.

## Conclusions

Results of calculating the medical cost of MS in Iran in 2019–2020 showed a significant burden on the Iranian health care system and patients, among which the medication cost had the largest share, which requires serious attention of health system policymakers in order to control the increasing cost along with paying attention to completing insurance coverage of interventions such as rehabilitation services for increase efficiency and social justice.

## Data Availability

All data obtained during this study is included in this article. The datasets used and/or analysed during the current study are available from the corresponding author on reasonable request.

## Abbreviations

IHIO: Iran Health Insurance Organization
COI: Cost of Illness
MS: Multiple sclerosis
RR: Relapsing-Remitting
PP: Primary Progressive
SP: Secondary Progressive
DMTs: Disease Modifying Therapies
EDSS: Expanded Disability Status Scale
